# An essential microRNA maturing microprocessor complex component DGCR8 is up-regulated in colorectal carcinomas

**DOI:** 10.1007/s10238-013-0243-8

**Published:** 2013-06-18

**Authors:** Bora Kim, Jae-ho Lee, Jong Wook Park, Taeg Kyu Kwon, Seong Kyu Baek, Ilseon Hwang, Shin Kim

**Affiliations:** 1 Department of Immunology, School of Medicine, Keimyung University, 1095 Dalgubeoldaero, Dalseo-Gu, Daegu, 704-701 South Korea; 2 Department of Pathology, School of Medicine, Keimyung University, 1095 Dalgubeoldaero, Dalseo-Gu, Daegu, 704-701 South Korea; 3 Department of Anatomy, School of Medicine, Keimyung University, 1095 Dalgubeoldaero, Dalseo-Gu, Daegu, 704-701 South Korea; 4 Department of Surgery, School of Medicine, Keimyung University, 1095 Dalgubeoldaero, Dalseo-Gu, Daegu, 704-701 South Korea

**Keywords:** MicroRNA biogenesis, Colorectal cancer, DGCR8, AGO2

## Abstract

MicroRNAs (miRNAs) regulate gene expression through degradation and/or translational repression of target mRNAs. Dysregulations in the miRNA machinery may be involved in carcinogenesis of colorectal cancer (CRC). The purpose of the current study was to evaluate the DiGeorge syndrome critical region gene 8 (DGCR8) and argonaute 2 (AGO2) mRNA expression in CRC and to evaluate the value of clinical parameters on their expression. We investigated the mRNA expressions of DGCR8 and AGO2 in 60 CRC tissues and adjacent histologically non-neoplastic tissues by using quantitative real-time PCR. Our study revealed that the mRNA expression level of DGCR8 is up-regulated in CRC. However, AGO2 mRNA expression was not significantly altered in CRC tissues. Neither DGCR8 nor AGO2 mRNA expression level was not associated with any clinical parameters, including age, tumor stage, CEA titer, and BMI in CRC cases. However, the mRNA expression levels of DGCR8 and AGO2 were positively correlated to each other. This study demonstrated for the first time that the DGCR8 mRNA expression level was up-regulated in CRC, suggesting its important role in pathobiology of colorectal carcinogenesis.

## Introduction

Colorectal cancer (CRC) is a malignant tumor that originates from the epithelium of colon and rectum and the third most common incident cancer among men worldwide [1]. It has been reported that the five leading primary cancer sites were the stomach, colon and rectum, lung, liver, and prostate in male during 2009 in Korea [2]. In Korea, annual percentage changes of CRC incidence in age-standardized incidence rates were 6.8 % in men between 1999 and 2009 using the world standard population as a standard population [2]. The pathogenesis of CRC is intricate and tightly regulated mechanisms, which involve the accumulation of both genetic and epigenetic alterations in the proliferating cells [3].

In recent years, gradually accumulating evidences have demonstrated that a wide range of biological processes such as cellular development, differentiation, proliferation, cell death, metabolism, and carcinogenesis are associated with a group of endogenous, small (approximately 17 nucleotides), and noncoding RNAs called microRNAs (miRNAs) [4–6]. The biogenesis of miRNA occurs in a well-organized process, referred to as the “miRNA machinery” [7]. The microprocessor complex mediates intranuclear biogenesis of precursor miRNAs from the primary miRNA transcript. Extranuclear, mature miRNAs are incorporated into the RNA-induced silencing complex (RISC) before interaction with complementary target mRNA that leads to protein translational repression or mRNA destabilization [8, 9]. The DiGeorge syndrome critical region gene 8 (DGCR8) is a part of microprocessor complex and has been shown to be essential for miRNA maturing [10]. The argonaute 2 (AGO2) protein is a constituent of a complex protein designated as RISC [11]. Previous study has demonstrated that DGCR8 mRNA expression level is down-regulated in prostate cancer [12]. Up-regulated mRNA expression level of DGCR8 has been revealed in epithelial skin cancer [8] and pleomorphic adenomas of the salivary gland [13]. It has been reported that the AGO2 mRNA expression level is up-regulated in epithelial skin cancer [8]. Though Papachristou et al. [7] studied the mRNA expression levels of Dicer, Drosha, and AGO2 in CRCs, there are little literatures about the mRNA expression level of DGCR8 and clinicopathologic association in the cancers.

In the present study, we aimed to investigate the mRNA expression levels of DGCR8 and AGO2 in human CRC tissues and corresponding adjacent non-neoplastic tissues from male patients with same cancer, and examined the correlation of the mRNA levels of these miRNA machinery components with various clinicopathologic parameters, including age, tumor stage, BMI, and CEA titer.

## Materials and methods

### Patients and tissues

Altogether, sixty male patients diagnosed with CRC were included in the study. Colorectal adenocarcinomas and adjacent non-neoplastic tissues were obtained from the patients undergoing surgery in Dongsan Medical Center (Daegu, Korea) between April 2008 and January 2010. Tissue samples were immediately frozen in liquid nitrogen and stored at −80 °C until RNA isolation. Tissue samples were provided from Keimyung Human Bio-resource Bank, Korea. All patients were explained the study purpose, and informed consent was obtained from each study participant. The protocols were approved by the Institutional Review Board of Keimyung University Dongsan Medical Center (approval #12–41).

### RNA and quantitative real-time PCR

Total cellular RNA was extracted from tissues using the TRIzol reagent (Molecular Research Center Inc., Cincinnati, OH, USA). RNA was quantified using Nanodrop 1000 (Thermo Scientific, Wilmington, Denmark). Each cDNA was synthesized form 2 μg of total RNA using M-MLV reverse transcriptase (Promega, Madison, WI, USA) according to the manufacturer’s protocol. By using the specific primer pairs described in Table 1 and SYBR GREEN Premix (Toyobo, Japan), quantitative real-time PCR (qPCR) was performed on the LightCycler^®^ 480 real-time PCR system (Roche Diagnostics, Mannheim, Germany). *β*-Actin was used as a housekeeping gene for normalization, and a no template sample was used as a negative control.

**Table 1:** Primer sequences of miRNA machinery components used in quantitative PCR

Components	Position	Sequence
AGO2	Forward	5′-TCATGGTCAAAGATGAGATGACAGA-3′
	Reverse	5′-TTTATTCCTGCCCCCGTAGA-3′
DGCR8	Forward	5′-CAAGCAGGAGACATCGGACAAG-3′
	Reverse	5′-CACAATGGACATCTTGGGCTTC-3′
*β*-Actin	Forward	5′-CAGCCATGTACGTTGCTATCCAGG-3′
	Reverse	5′-AGGTCCAGACGCAGGATGGCATG-3′

### Statistical analysis

Statistical analysis was performed with SPSS 18.0 (SPSS Inc., Chicago, IL, USA). Statistical comparisons for significance were made with Wilcoxon signed-rank test for paired samples. Differences between the groups were analyzed statistically by using Student’s *t* test. The correlations between *DGCR8* and *AGO2* expressions and clinicopathologic parameters were assessed with the Pearson’s correlation coefficient analysis. Generally, *P* value of <0.05 was established to denote significance in all statistical analyses performed in the study.

## Results

### Expression levels of DGCR8 and AGO2 mRNA in colorectal cancer tissues and adjacent non-neoplastic colorectal tissues of CRC patients

**Fig. 1:**
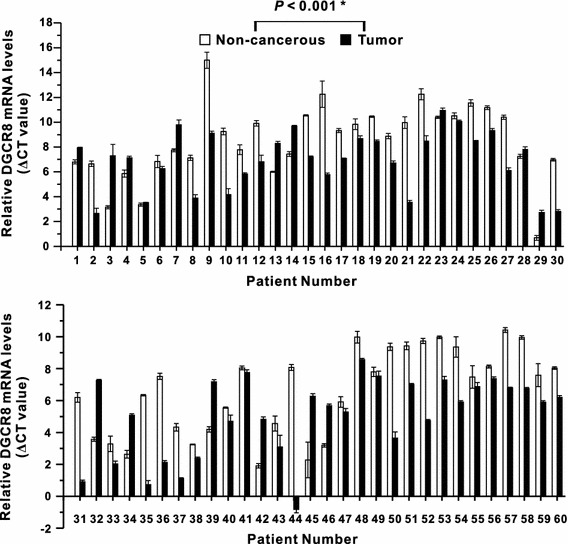
The relative DGCR8 mRNA level (normalized to the corresponding *β*-actin mRNAs) in tumor tissues compared to adjacent non-cancerous colorectal tissues. *Asterisk* indicates Wilcoxon signed-rank test

**Fig. 2:**
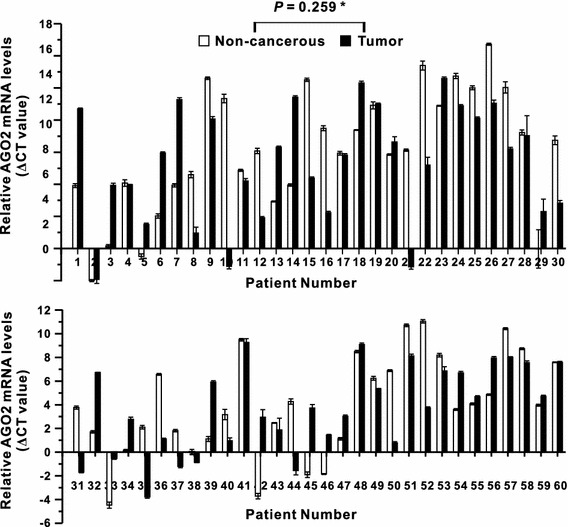
The relative AGO2 mRNA level (normalized to the corresponding *β*-actin mRNAs) in tumor tissues compared to adjacent non-cancerous colorectal tissues. *Asterisk* indicates Wilcoxon signed-rank Test

**Fig. 3:**
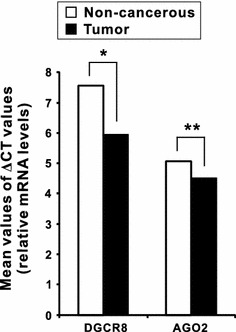
Relative DGCR8 and AGO2 mRNA expression in CRC group and in the control group; *P < 0.001; **P = 0.26

The mRNA expression levels of DGCR8 and AGO2 were quantified by qPCR in paired specimens of human cancerous colorectal tissues and their respective non-neoplastic colorectal tissues from 60 patients with CRC. The DGCR8 and AGO2 mRNA levels were normalized to the level of *β*-actin mRNA. Then, the qPCR data were analyzed by using ΔCT values [14]. Our study revealed that DGCR8 mRNA expression was significantly higher in carcinomatous tissues than in the corresponding non-neoplastic tissues in 44 of the 60 patients with CRC (*P* < 0.001; Fig. 1). However, AGO2 mRNA expression was not significantly altered (*P* = 0.259, Fig. 2). The mean value of DGCR8 mRNA expression level in cancerous tissues was significantly higher than in non-neoplastic colorectal tissues (*P* < 0.001); however, the mean value of AGO2 mRNA expression level was not different between cancerous and non-neoplastic tissues (Fig. 3).

### Relationship between DGCR8 and AGO2 mRNA expression levels and the clinical parameters in patients with CRC

The recent study demonstrated that AGO2 is not associated with clinicopathological features, including patient age, gender, and overall survival or tumor location, grade, stage, and size, in CRC [7]. Therefore, we investigated whether the mRNA expression levels of DGCR8 and AGO2 are associated with any clinicopathologic parameters of CRC. Prior to statistical analysis, raw qPCR data of DGCR8 and AGO2 mRNA expression levels were normalized to reference gene, *β*-actin. Then, the qPCR data were analyzed by the 2^−ΔΔCT^ method [14]. To evaluate the influence of the clinical parameters on mRNA expression of DGCR8 and AGO2, patients were classified according to each clinical characteristic. The clinicopathologic parameters in 60 patients (mean age: 63.6 ± 10.4 years) with CRC according to DGCR8 and AGO2 mRNA expression levels were presented in Table 2. Unfortunately, DGCR8 and AGO2 mRNA expression levels were not statistically associated with age, tumor stage (TNM), CEA titer, and BMI clinical parameters in our CRC specimens. However, higher mRNA expression level of DGCR8 was found in higher group of AGO2 mRNA expression level, and vise versa. Therefore, the mRNA expression levels of DGCR8 and AGO2 were positively correlated to each other.

**Table 2:** Correlation of the clinicopathologic parameters with DGCR8 and AGO2 mRNA expression levels in CRCs

Variables	Total	AGO2	*P*	DGCR8	*P*
Total	60 (100)	29 (48.3)		39 (65.0)	
Age			0.631		0.337
≤50	25 (41.7)	13 (52.0)		18 (72.0)	
>50	35 (58.3)	16 (45.7)		21 (60.0)	
BMI			0.901		0.976
≤25	43 (71.7).	21 (48.8)		28 (65.1)	
>25	17 (28.3)	8 (47.1)		11 (64.7)	
T stage			0.970		0.713
T1	4 (6.7)	2 (50.0)		3 (75.0)	
T2	9 (15.0)	4 (44.4)		5 (55.6)	
T3	36 (60.0)	17 (47.2)		25 (69.4)	
T4	11 (18.3)	6 (65.5)		6 (54.5)	
N stage			0.504		0.405
N0	33 (55.0)	14 (42.4)		21 (63.6)	
N1	16 (26.7)	9 (56.3)		10 (62.5)	
N2	10 (16.7)	6 (60.0)		8 (80.0)	
N3	1 (1.7)	0 (0)		0 (0)	
M stage			0.269		0.664
Negative	56 (93.3)	26 (46.4)		36 (64.3)	
Positive	4 (6.7)	3 (75.0)		3 (75.0)	
CEA (ng/ml)			0.282		0.309
≤5	47 (78.3)	21 (44.7)		29 (61.7)	
>5	13 (21.7)	8 (61.5)		10 (76.9)	
AGO2					**<0.001**
High	29 (48.3)	–		27 (93.1)	
Low	31 (51.7)	–		12 (38.7)	
DGCR8			**<0.001**		
High	39 (65.0)	27 (69.2)		–	
Low	21 (35.0)	2 (9.5)			

Significant bold values indicate statistical analysis

*DGCR8* DiGeorge syndrome critical region gene 8, *AGO2* argonaute 2

## Discussion

MicroRNA pathway is involved in the regulation of various cellular processes, including cellular development, differentiation, proliferation, cell death, metabolism, and carcinogenesis [4–6]. Deregulation of miRNAs in various cancers may be related with altered expression and function of the genes involved in the miRNA machinery components, including DGCR8 [13] and AGO2 [15].

The objectives of this study were to investigate the mRNA expression levels of DGCR8 and AGO2 by RT-qPCR method in pair-matched colorectal specimens and analyze their correlation with different clinical characteristics. We therefore identified the mRNA expression levels of DGCR8 and AGO2 in CRC tissue compared with adjacent non-neoplastic colorectal tissue in 60 patients with CRC. We determined that DGCR8 mRNA expression level was up-regulated in CRC. Just like the results of our experiment, Sand et al. [8] demonstrated that DGCR8 mRNA expression level was up-regulated in epithelial skin cancers. However, Shaikhibrahim et al. [12] showed also its down-regulation in prostate cancer. DGCR8 is a cofactor for Drosha, an RNAse III endonuclease, and also a part of the microprocessor complex and has been found to be essential for miRNAs maturation [10]. Drosha and DGCR8 have evolved to regulate each other via a complicated double-negative feedback circuit in which DGCR8 stabilizes Drosha through a direct interaction [16]. It thus would be needed to assess the correlation between DGCR8 and Drosha mRNA expression levels in CRC cases. Interestingly, we found the significant association between DGCR8 and AGO2 mRNA expression levels in CRC. This result suggested that DGCR8 and AGO2 may be associated with colorectal carcinogenesis together.

Recently, rapidly accumulating evidence has been shown that perturbation in miRNA biogenesis is closely associated with development and progression of a variety of cancers, including CRC [7, 17, 18]. As one of key enzymes in the miRNA generating process, DICER and DROSHA have been frequently studied [19–25]; however, there was a little study about DGCR8 and AGO2 [8, 26]. Additionally, DGCR8 and AGO2 mRNA expression levels analysis did not even show any significant differences between malignant melanomas (primary cutaneous malignant melanoma and cutaneous malignant melanoma metastases) and benign melanocytic nevi [26]. Nevertheless, because DGCR8 and AGO2 are two important components in miRNA maturation, we investigated whether the altered mRNA expression levels of DGCR8 and AGO2 are associated with the carcinogenesis of CRC. As shown in Table 2, no association between altered expressions of the two miRNA machinery components and clinical parameters, including age, tumor stage, CEA titer, and BMI, was revealed. Our result, in agreement with recent study [7], showed no association between DICER, DROSHA, and AGO2 and clinicopathological characteristics. Due to a short follow-up period, we could not assess the prognostic value of each miRNA machinery component in our study group. Therefore, further investigation with longer follow-up period will resume, and prognostic impact of the components will be analyzed as soon as possible.

In this study, we investigated the mRNA expression levels of two selected miRNA machinery components, DGCR8 and AGO2, and their clinical association in CRCs for the first time. Our data demonstrated that DGCR8 is significantly up-regulated in CRC, suggesting that reduced expression of DGCR8 may play an important role during the process of colorectal carcinogenesis. Considering deep correlation between DGCR8 and AGO2 in CRCs, further study of these miRNA components should be needed in various colorectal neoplastic regions.
